# The developing adolescent brain: Implications for youth justice and children in criminal proceedings

**DOI:** 10.1016/j.dcn.2026.101807

**Published:** 2026-09-02

**Authors:** Sarah-Jayne Blakemore

**Affiliations:** Department of Psychology, University of Cambridge, UK

**Keywords:** Juvenile justice, Youth justice, Teenage brain, Minimum age of criminal responsibility, Joint enterprise, Peer influence, Criminal justice system

## Abstract

Adolescence is a unique developmental stage, defined by neural reorganisation and a heightened sensitivity to the social environment. Brain maturation is a prolonged process extending well into the twenties, involving microstructural refinements such as axonal myelination and synaptic pruning, which are partly dependent on environmental input. This period of heightened neuroplasticity coincides with a shift toward peer affiliation and increased susceptibility to risk-taking, particularly in social settings. Consequently, adolescent decision-making should be viewed through a social lens, in which the drive to avoid social exclusion and to secure peer acceptance acts as a powerful motivator of behaviour. This framework underscores the importance of social connections and suggests that, conversely, disruptions to these relationships, and in particular social isolation, can be harmful to adolescent development. Studies have demonstrated that even brief periods of social isolation in adolescence can increase reward seeking and reward learning, heighten threat sensitivity and reduce risk perception. The research reviewed in this paper has implications for how young people are treated in the youth justice system. I argue that justice systems should evolve from punitive approaches toward rehabilitative frameworks and age-appropriate legal thresholds grounded in evidence-based insights into the developing adolescent brain and mind.

## 1 Introduction

There is nothing new about the attributes and behaviours we tend to associate with adolescents. Over 2000 years ago, Aristotle was to have said of youth: ‘They are passionate, hot-tempered, and carried away by impulse… At this age more than any other they are fond of their friends and companions…’ (Aristotle, trans. 1954). The transitional period of adolescence, extending from approximately 10–24 years (Sawyer et al., 2018), has long been characterised as a period of heightened impulsivity, volatility, proneness to risk-taking and affiliation with peers.

What is relatively new is our understanding of how these characteristics unfold in the adolescent brain and mind. Over the past three decades, developmental cognitive neuroscience studies have established that the human brain continues to mature throughout childhood and adolescence, and well into early adulthood. This protracted development occurs alongside characteristic adolescent behaviours, including increased risk-taking, a gradual improvement in self-regulation and a shift in social orientation towards peers (Blakemore and Mills, 2014).

This paper begins by reviewing the evidence for extended brain development and heightened neuroplasticity. The second section describes how adolescent neural changes are accompanied by changes in cognitive processing and decision-making, particularly in social contexts. Disruptions to social connection can be detrimental in adolescence, and the third section describes the effects of social isolation on reward and threat learning, reward seeking and risk perception. Together, these findings highlight how the social environment can interact with neurocognitive development to shape adolescent behaviour and decision-making. The fourth section explores the implications of this body of evidence for the youth (also called juvenile) justice system and reflects on how developmental cognitive neuroscience can inform legal policies that align with contemporary understanding of adolescent development.

## 2 Section 1: Development of the brain in adolescence

Our understanding of adolescent brain development has been informed by longitudinal structural magnetic resonance imaging (sMRI) studies. These studies have consistently demonstrated protracted changes in both grey and white matter volume throughout adolescence and into early adulthood (Mills et al., 2016, Tamnes et al., 2017, Mousley et al., 2025). Average cortical grey matter volume typically follows an inverted U-shaped trajectory, reaching a peak in late childhood, before undergoing a sustained decline that stabilises in the mid- to late twenties (Bethlehem et al., 2022p, Giedd et al., 1999, Mills et al., 2016, Sowell et al., 2004). In parallel with grey matter development, cerebral white matter volume increases linearly between childhood and early adulthood (Lebel and Beaulieu, 2011, Tamnes et al., 2017, Bethlehem et al., 2022p). A recent study of brain network topology identified a turning point around age 9, followed by continued non-linear changes in structural connectivity that persist until around age 32 (Mousley et al., 2025). These findings highlight the prolonged and complex nature of human brain development.

Animal models and post-mortem human brain tissue studies suggest that these macrostructural changes reflect several key microstructural developmental processes. The increase in white matter is primarily driven by ongoing myelination, where insulating myelin sheaths wrap around axons to increase the speed and fidelity of signal transmission (Fields, 2005). In parallel, axons gradual increases in diameter, further increasing signal processing efficiency (Giorgio et al., 2010, Palmer et al., 2022, Dipnall et al., 2024). Myelination and axonal growth contribute to the increase in white matter volume and concomitant decrease in grey matter volume observed in structural MRI studies. Diffusion Tensor Imaging studies support this by showing age-related increases in fractional anisotropy, a microstructural index associated with greater axon packing, myelination and fibre coherence (Giorgio et al., 2010, Lebel and Beaulieu, 2011).

Another microstructural process that occurs during development involves the reorganisation of synapses. Our understanding of synaptic development was initially informed by the pioneering studies of Peter Huttenlocher. Using electron microscopy to count synaptic density in post-mortem human brain tissue at different ages, Huttenlocher demonstrated that cortical regions undergo an initial period of exuberant synaptogenesis (an overproduction of connections), followed by synaptic pruning, the selective elimination of redundant or less-used synapses (Huttenlocher and Dabholkar, 1997). Synaptic pruning increases the signal-to-noise ratio and enhances the efficiency and specificity of neural circuits. Huttenlocher’s work further demonstrated that the timing of these changes is heterogeneous across the brain. While synaptic pruning in sensory and motor cortices is completed in childhood, synaptic density in parts of the prefrontal cortex continues to increase until late childhood, before gradually declining throughout adolescence (Huttenlocher, 1979).

**Fig. 1:**
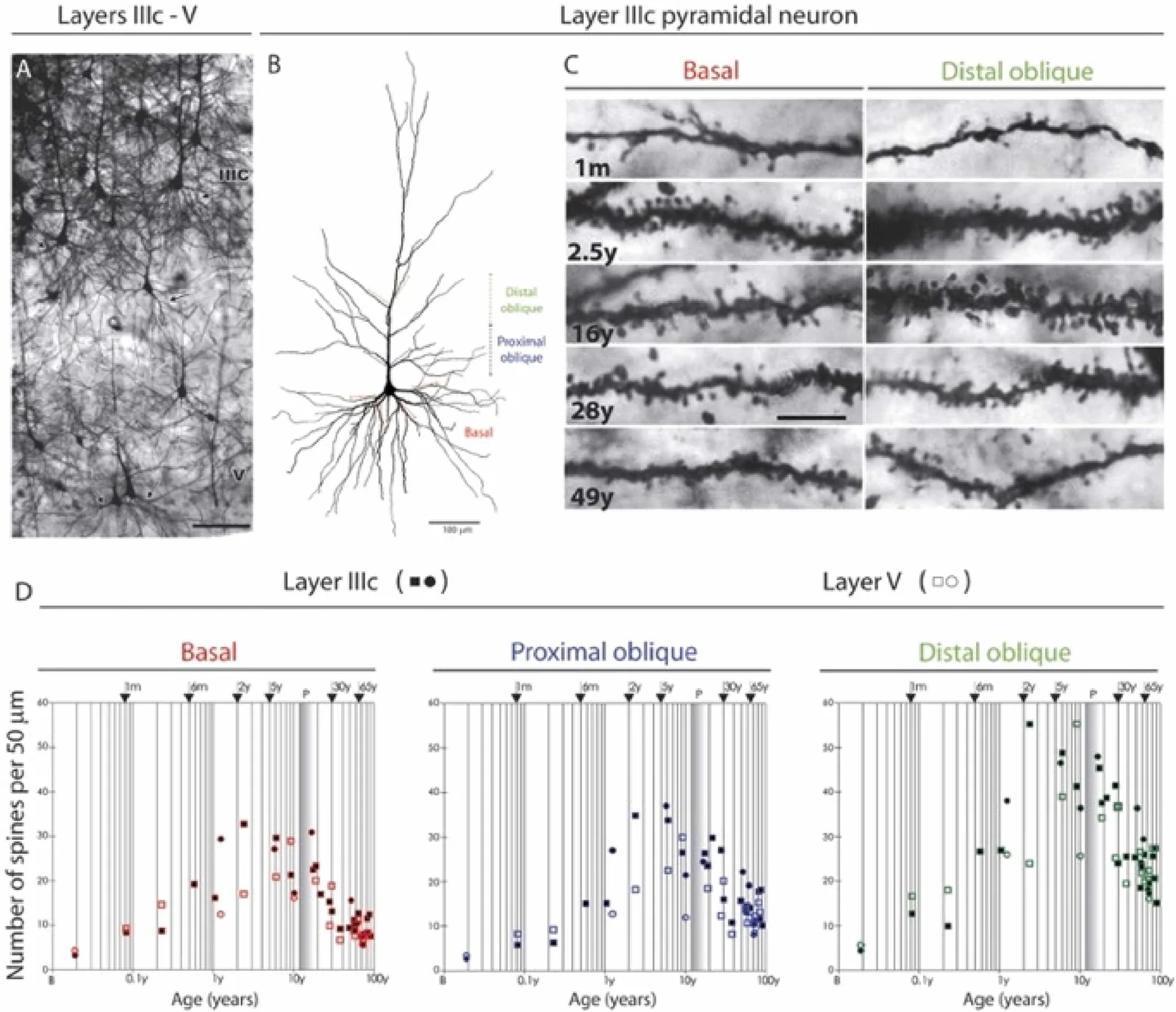
Synaptic development in human prefrontal cortex. (A) Micrograph of human cortex from post-mortem tissue. (B) Reconstruction of a layer IIIc pyramidal neuron showing basal, proximal oblique and distal oblique dendrites. (C) Example dendritic segments across age, illustrating changes in spine density. (D) Spine density plotted against log-age for layer IIIc and layer V neurons across dendritic compartments, showing extended growth in childhood, followed by protracted pruning into adulthood. From Petanjek et al. (2011).

Huttenlocher’s pioneering studies relied on tissue from a relatively small number of post-mortem brains, and only one 19-year-old brain in the range between 15 and 32 years. However, his findings were replicated and expanded by Petanjek and colleagues, who investigated synaptic density in 32 post-mortem brains ranging in age from one week to 91 years (Petanjek et al., 2011). This study confirmed a prolonged phase of dendritic growth and synaptic proliferation in the prefrontal cortex that persists until late childhood, followed by a protracted decline in synapses throughout the second and third decade of life (see Fig. 1).

The refinement of synapses, combined with myelination of axons, creates a neurobiological state optimised for integrating environmental experience (Fields, 2005, Wiesel and Hubel, 1963). Synaptic pruning is not a random process; it is an activity-dependent, selective elimination driven by the frequency and consistency of neuronal communication. Synapses that are frequently and consistently activated by environmental inputs are strengthened and retained (Huttenlocher and Dabholkar, 1997). Conversely, synapses that are rarely activated are more likely to be pruned. This competition among synapses for survival is regulated by chemical signals from glial cells, particularly microglia and astrocytes, which clear away redundant connections (Tremblay et al., 2011, Chung et al., 2013). During development, the environment can determine which neural circuits are reinforced and which are pruned away, effectively encoding an individual’s experience into their neural architecture. As such, adolescence can be viewed as a sensitive period of ‘adaptive plasticity’ (Casey et al., 2025, Fuhrmann et al., 2015, Wilbrecht and Davidow, 2024), a window of opportunity in which environmental input shapes brain development (Larsen and Luna, 2018). This heightened neuroplasticity endows adolescents with an increased capacity for learning, adaptation and positive change, but it also confers vulnerability, if the environment is negative or stressful.

As found in Huttenlocher’s post-mortem brain tissue studies, human structural MRI studies have shown that different parts of the brain develop at different rates. In general, posterior regions develop earlier than anterior regions. The occipital (visual) cortex and other sensory areas undergo most of their structural development early in life and change relatively little after the early teenage years (Tamnes et al., 2017). In contrast, regions within the parietal, temporal and frontal cortices follow a more extended developmental trajectory.

**Fig. 2:**
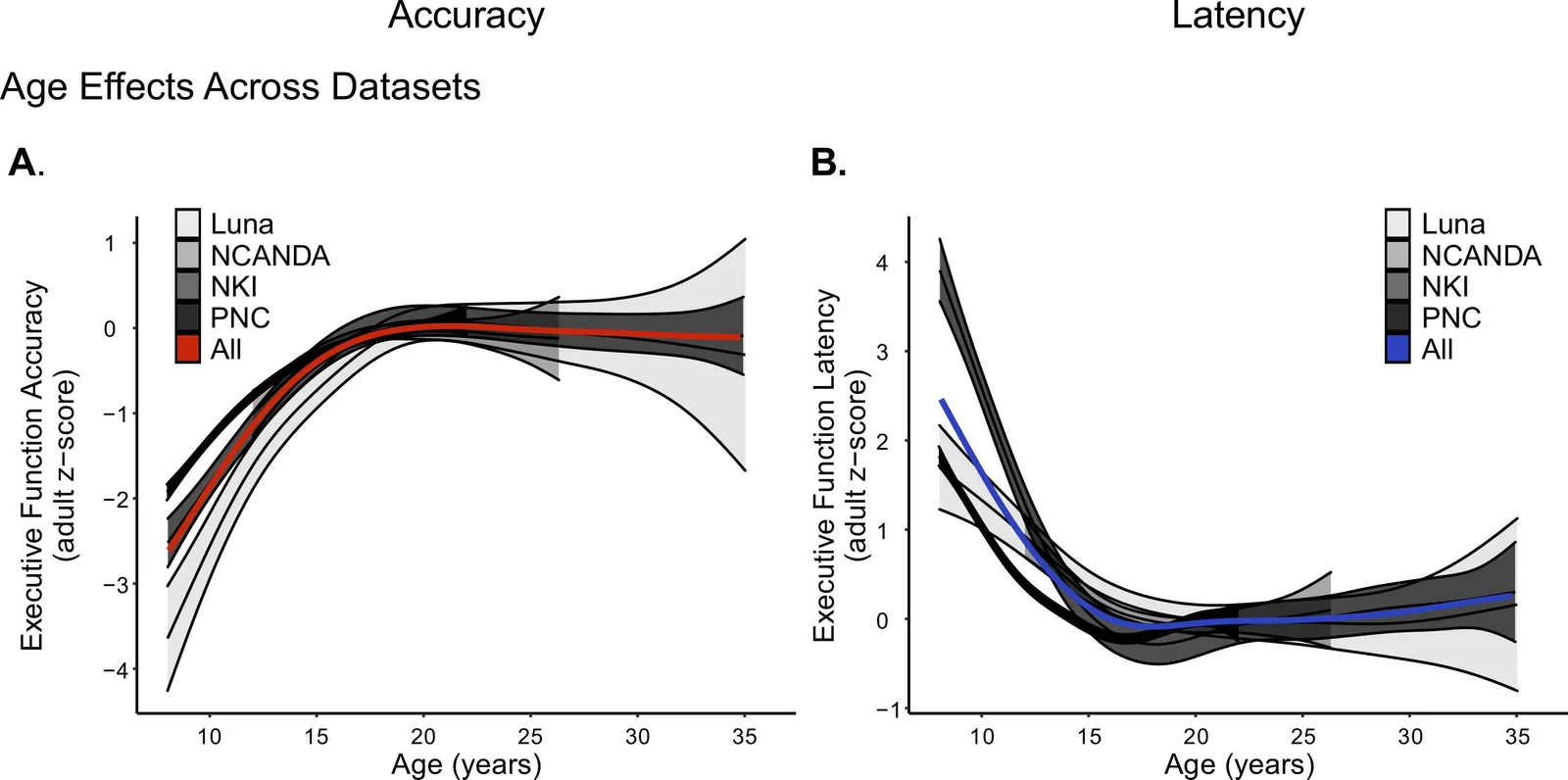
Developmental trajectories of executive function. Age-related changes in executive function accuracy (left) and latency (right) from 8 to 35 years across datasets (Luna, NCANDA, NKI, PNC) and the aggregate (All). Accuracy increases and latency decreases with age, with rapid changes in early adolescence and stabilisation by the early twenties. From Tervo-Clemmens et al. (2023).

The later maturing regions, including the prefrontal cortex, support higher-order executive functions such as planning, self-control and decision-making. Performance on tasks that rely on the prefrontal cortex, including inhibitory control, improves during adolescence and stabilises in the early to mid-twenties, broadly mirroring the prolonged structural development of prefrontal regions (Tervo-Clemmens et al., 2023) (see Fig. 2). For example, studies using response inhibition paradigms such as the antisaccade task, in which participants see a visual target appear suddenly to one side of the screen and are instructed not to look directly at it, but instead to make an eye movement (saccade) to the opposite side, show age-related improvements in the ability to suppress reflexive responses. There are gradual reductions in errors and decreases in reaction times in these tasks between childhood and the early twenties (e.g. Luna et al., 2008; Parr et al., 2022).

The development of the prefrontal cortex is more protracted than that of limbic regions, including the amygdala and ventral striatum (note there are substantial individual differences in the relative developmental trajectories of these regions; Mills et al., 2014). These regions drive emotional and motivational responses and reward processing, and are especially sensitive to rewarding and emotional stimuli in adolescence (Hare et al., 2008). The developmental imbalance between the hypersensitive limbic system and the late-developing prefrontal cortex has been proposed to contribute to difficulties inhibiting responses to emotionally salient information and heightened risk-taking, particularly in social contexts (Heller and Casey, 2016).

## 3 Section 2: Adolescence as a sensitive period of social reorientation

**Fig. 3:**
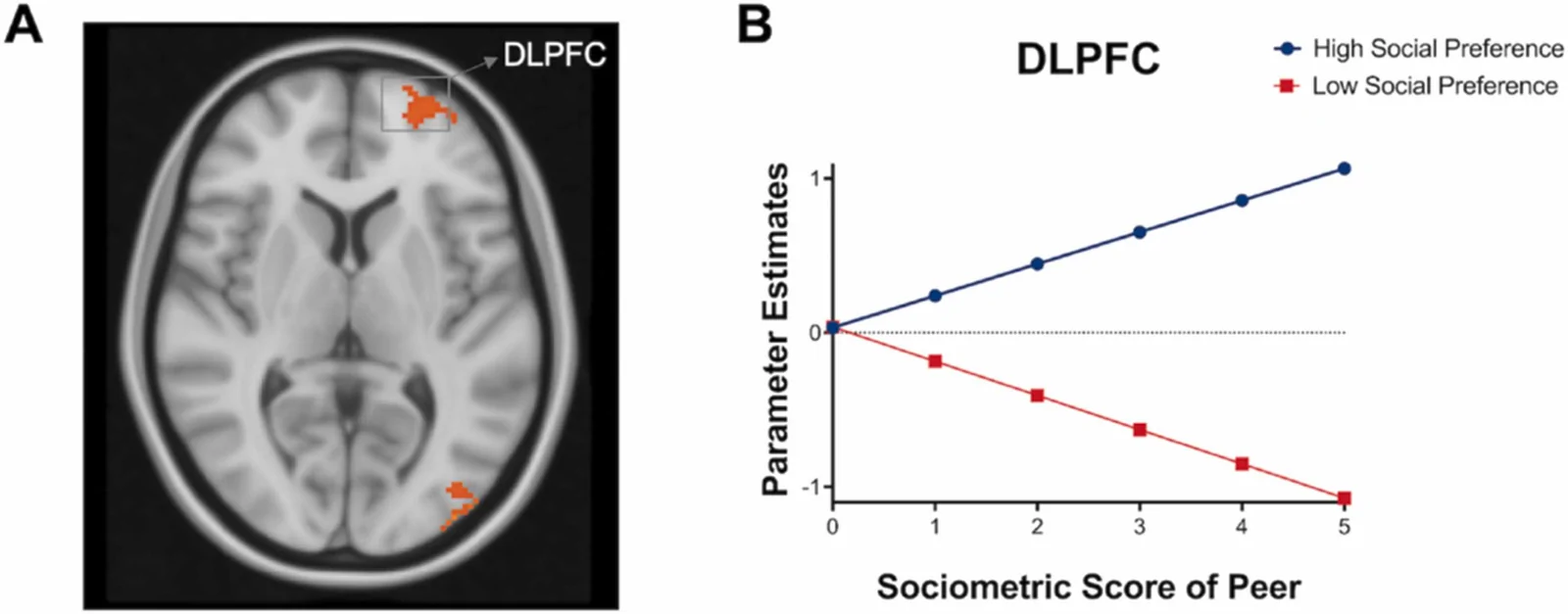
Dorsolateral prefrontal cortex activity and peer preference. (A) Task-related activity in the dorsolateral prefrontal cortex when viewing faces of peers. (B) Activity in the dorsolateral prefrontal cortex was differentially activated when tracking peers who had been classified as high versus low in social preference. From Dai et al. (2023).

Adolescence is a period of social reorientation, during which young people become increasingly attuned to the attitudes and influence of their peer group (Blakemore and Mills, 2014). Time spent with friends increases, and an adolescent’s evaluation of their social and personal worth becomes progressively contingent upon peer perception. This psychological transition occurs alongside significant changes in the social environment. The move from small primary schools to larger secondary schools in early adolescence introduces expansive, unstable social hierarchies. Social networks undergo substantial reorganisation during adolescence, with relationships becoming increasingly reciprocal and adolescents becoming better able to navigate and maintain complex social relationships across development (Burnett Heyes et al., 2015, Escribano et al., 2023). Recent functional neuroimaging research has investigated how the adolescent brain navigates real world social networks. Activity in the fusiform face area and the dorsolateral prefrontal cortex when viewing faces of peers was associated with social preference (likability; Dai et al., 2023). The authors suggested that, while the fusiform face area may be involved in allocating attention to both rewarding and threatening group members, the dorsolateral prefrontal cortex supports behavioural regulation and goal-directed attention toward socially relevant individuals (see Fig. 3). Collectively, these findings indicate that the adolescent brain is attuned to social hierarchies, employing specialised circuits to navigate complex social environments and influence social interactions and decision making.

Peer influence on decision-making is particularly pronounced during adolescence, as sensitivity to peer approval and inclusion increases (Steinberg and Monahan, 2007, Blakemore and Mills, 2014). For example, adolescent-typical risk-taking such as smoking, substance use and dangerous driving, is more likely to occur in the presence of friends than when alone. Data from the American Automobile Association Foundation for Traffic Safety indicates that the risk of fatal car accidents for drivers under 21 increases by 44% with one passenger and quadruples with three or more passengers (Tefft et al., 2013). Experimental studies corroborate these real-world observations. In a simulated driving task, age-related differences in risk-taking were not present when participants performed the task on their own (Gardner and Steinberg, 2005), suggesting that in relatively neutral (‘cold’) cognitive contexts, free from emotional interference or social cues, adolescents can possess adult-like capacities for risk assessment. However, the presence of peers (a ‘hot’ context) altered this developmental pattern: while adults’ risk-taking behaviour was unaffected by peers, adolescents demonstrated a significant increase in risky driving when friends were present. Notably, the presence of an adolescent’s mother had the opposite effect: risk-taking in 14-year-olds was reduced when their mother was present relative to when they were alone (Telzer et al., 2015). This indicates that adolescent risk-taking in social contexts is not only a consequence of distraction by another person and might be linked specifically to the presence of peers, perhaps due to the heightened value placed on the approval of peers.

This prioritisation of peers’ opinions over adults’ is also evident in the perception of potentially risky situations. While most age groups were more influenced by adults’ risk ratings, young adolescents (aged 12–14) were more influenced by the opinions of other teenagers than by those of adults, and mid-adolescents (aged 15–18) were equally influenced by teenagers and adults (Knoll et al., 2015, Knoll et al., 2017). This shift may be driven by a hypersensitivity to social exclusion in adolescence. Studies have demonstrated that the experience of (purportedly) being excluded by others in an online game more strongly activates regions associated with affective distress, such as the anterior cingulate cortex, and results in greater decreases in mood, in adolescents compared with adults (Masten et al., 2009, Sebastian et al., 2010).

The heightened reactivity to social exclusion suggests that adolescent conformity to peers might be a socially rational response to developmental priorities (Blakemore, 2018). For an adolescent, the immediate social risk of declining a peer-led activity, and the subsequent threat of ostracism, may be perceived as more consequential than the abstract, long-term health or legal risks associated with the behaviour itself. Thus, what may appear to be a failure of self-control or a tendency towards bad decision making can be reinterpreted as a socially rational, strategic effort to avoid the emotional pain of social exclusion. By shifting the focus from a pathological deficit model, this framework conceptualises the peer-induced down-regulation of self-control not as a cognitive failure, but as an adaptive mechanism. In the presence of peers, prioritising immediate social status and affiliation can be seen as rational, serving an adaptive developmental function, even if it results in a heightened propensity for risk-taking.

Peer influence in adolescence is not exclusively negative. Adolescents can also motivate each other towards prosocial and health-promoting behaviours. Research suggests that adolescents are particularly susceptible to positive social influence. For example, the probability of being influenced by others’ charitable donations decreasing significantly from early adolescence into adulthood (Chierchia et al., 2020): young adolescents’ donations were more influenced by generous peers compared with adults’ donations. Furthermore, while adults exhibited ‘opportunistic conformity,’ by aligning more readily with selfish norms (reducing their donations when others gave less), adolescents showed no such bias, suggesting they may be more balanced in their susceptibility to both prosocial and selfish social influence.

This has also been demonstrated in experimental chat room paradigms. Young adolescents increased their intentions to volunteer and assist others after observing popular peers engage in similar prosocial behaviours (Choukas-Bradley et al., 2015). These positive shifts in intentions persisted to a certain extent when participants made subsequent decisions in private, suggesting that heightened social sensitivity during adolescence can support the internalisation of prosocial values. Another study showed that adolescents who received positive (but not negative) feedback from peers after making prosocial (generous) decisions in a public goods game increased their prosocial behaviour, speculatively because aligning with peers is experienced as intrinsically rewarding (van Hoorn et al., 2016).

This tendency towards prosocial conformity in adolescence can be harnessed to spread positive and prosocial norms. Public health campaigns may be more effective if they focus less on fear-based messaging about physical harm and instead leverage social norms to promote positive peer influence. Supporting this, a U.S.-based trial found that schools that were randomly assigned to peer-led anti-bullying programmes, where students designed and delivered anti-bullying campaigns themselves, showed a greater reduction in bullying and student conflict over the following year, compared to schools using teacher-led programmes (Paluck et al., 2016). This illustrates adolescents’ capacity to influence each other in positive ways and highlights the powerful role peers can play in shaping adolescent behaviour.

The quality of peer relationships during this period plays a crucial role in shaping psychological outcomes. Difficulties such as social rejection, bullying and loneliness are associated with an increased risk of mental health problems, including depression (Alsarrani et al., 2022, Arseneault, 2018, Platt et al., 2013, Schwartz-Mette et al., 2020), whereas supportive, high-quality friendships can promote resilience and protect against psychological difficulties (Alsarrani et al., 2022, van Harmelen et al., 2017, van Harmelen et al., 2021).

These findings highlight the importance of stable and meaningful social connections during adolescence and suggest that disruptions to, or the absence of, such relationships may have harmful consequences. Consistent with this, population-level research indicates that loneliness is particularly pronounced during this developmental stage: self-reported loneliness is more prevalent among young people aged 16–24 than in any other age group (Barreto et al., 2021) and has increased over the past 15 years (Twenge et al., 2021).

## 4 Section 3: Social isolation and loneliness in adolescence

Much has been learned about the effects of social isolation on brain and behavioural development from rodent studies (Matthews and Tye, 2019, Xiong et al., 2023, Orben et al., 2020). During adolescence (from approximately postnatal day 21 (PND 21) until around PND 60), rodents exhibit an orientation toward peers, actively seeking social play as a critical input for social learning and healthy maturation (Vanderschuren et al., 2016). Even brief periods of isolation (as short as 24 h) during adolescence can lead to behavioural disruptions, including increased anxiety, hyperactivity and a heightened sensitivity to social and chemical or alcohol rewards that may predispose the animal to long-term addiction (Ikemoto and Panksepp, 1992, Maisonnette et al., 1993, McCool and Chappell, 2009, Morley and Worsham, 1978). Chronic isolation (one week or longer) results in more severe and enduring consequences, including hyper-reactivity to stress and heightened aggression (Heidbreder et al., 2000, St Popova and Petkov, 1990). Furthermore, isolation during adolescence alters cognitive processes, including reward learning (Robbins, 2016), reward seeking (Novick et al., 2018), reversal learning (Amitai et al., 2014) and attention shifting (Schrijver and Würbel, 2001).

Social isolation in adolescent rodents also affects brain development, leading to dysregulation of dopamine signalling: dopamine release increases in reward-related regions such as the nucleus accumbens, while dopamine activity decreases within the prefrontal cortex (Novick et al., 2018, Hall, 1998). These effects are most pronounced in adolescence and are less evident when isolation occurs before weaning or in adulthood.

These findings suggest that peer interaction is a basic need in adolescent development. They also provide a mechanistic framework for understanding how social isolation might influence cognition and behaviour in humans.

## 5 Social isolation in human adolescents

Despite a lot of evidence from animal models, relatively little is known about how isolation affects development in human adolescents due to the lack of experimental studies. To address this gap, we conducted a within-participant experimental study to examine whether brief periods of social isolation influence behaviour and affect (Tomova et al., 2025). Adolescents aged 16–19 completed a set of behavioural tasks assessing reward processing, threat learning and risk evaluation under three conditions: baseline (a typical day), complete isolation and isolation with access to virtual interaction (including social media).

During the two isolation sessions, participants spent 3–4 h alone in a room containing basic amenities (refreshments, puzzles, a computer, access to a bathroom, a skylight) but no social contact. In the virtual interaction condition, participants spent the same amount of time alone in the same room but were permitted access to devices such as phones and tablets, enabling online communication. This design allowed us to test whether virtual forms of social connection mitigate the effects of physical isolation.

**Fig. 4:**
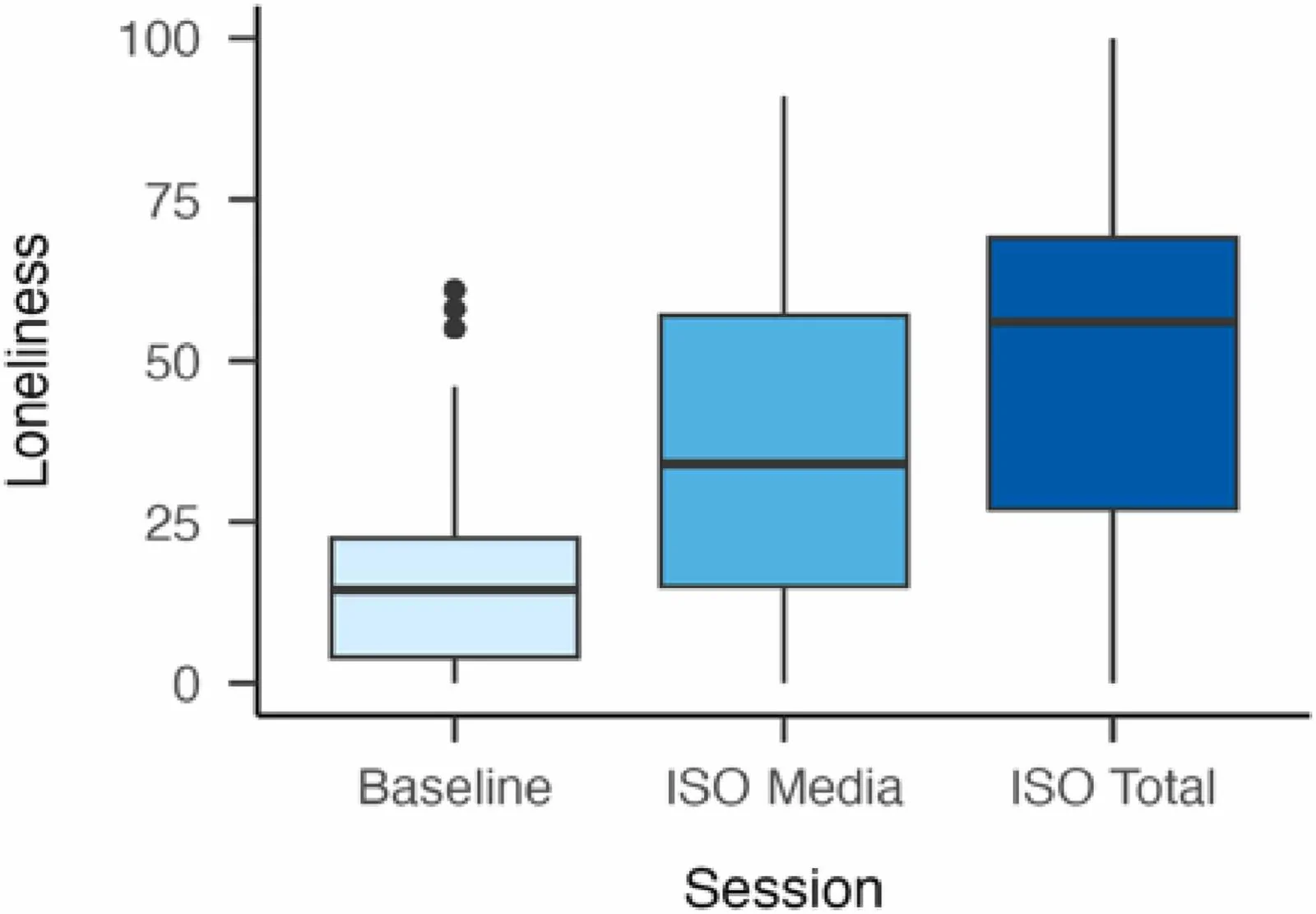
Self-reported loneliness after isolation. Loneliness scores were lowest at baseline, higher during the isolation session with access to virtual interaction and social media (ISO Media) and highest during the total isolation session (ISO Total). Boxplots display medians and interquartile ranges; individual points denote outliers. From Tomova et al. (2025).

**Fig. 5:**
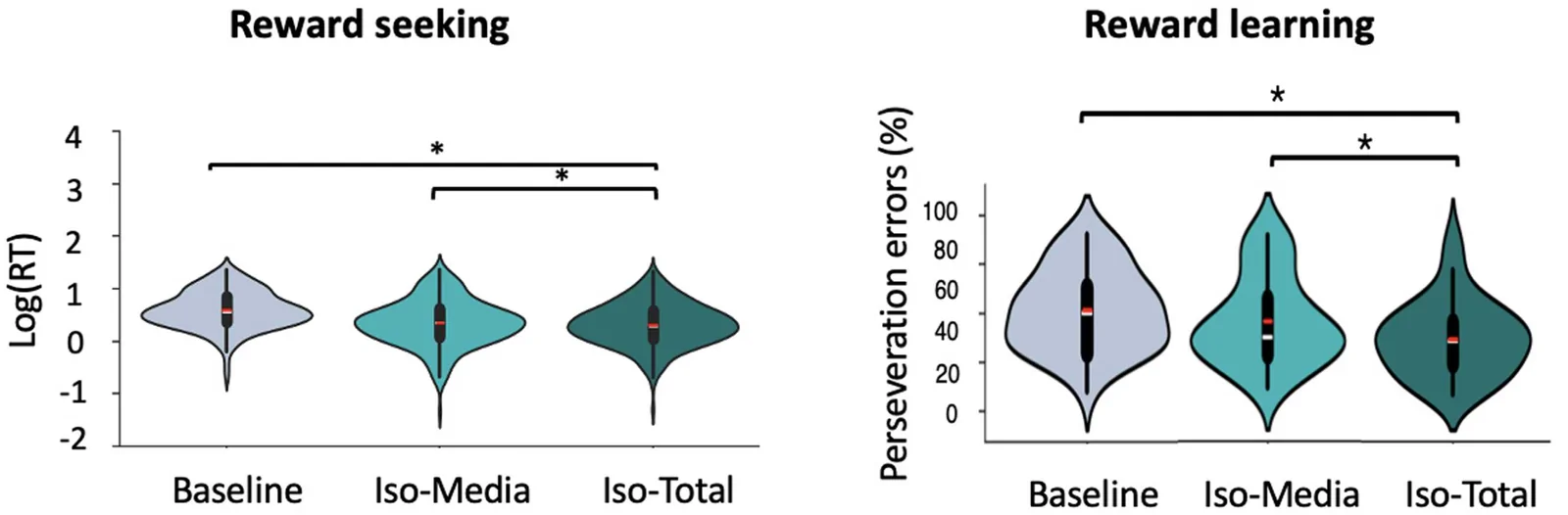
Impact of social isolation on reward seeking and reward learning. Violin plots show the distribution of log-transformed response times (log(RT)) during a reward seeking task and percentage perseverative errors in a reward learning task. Participants responded significantly faster in the reward seeking task and more accurately in the reward learning task after total isolation (Iso-Total) compared to both the Baseline and the virtual interaction and social media session (Iso-Media) conditions. From Tomova et al. (2025).

The results showed that total isolation increased self-reported loneliness and reduced positive mood (Tomova et al., 2025; see Fig. 4). Replicating findings from the rodent studies, total isolation also heightened reward sensitivity: adolescents made faster decisions to pursue rewards and showed more efficient learning of reward contingencies after total isolation compared with baseline and isolation with access to virtual interaction (Fig. 5). Notably, greater social-reward seeking was associated with higher levels of loneliness during isolation. Isolation also amplified threat learning. Using a Pavlovian conditioning paradigm, participants showed increases in both subjective anxiety and physiological arousal (electrodermal activity) toward threat-predictive cues (Towner et al., 2024). As was the case for social-reward seeking, the level of self-reported loneliness during the isolation session predicted the effects on threat learning.

These results suggest that loneliness was associated with increased sensitivity to both positive and negative cues, amplifying both approach-oriented (Tomova et al., 2025) and avoidance-oriented learning (Towner et al., 2024). In addition, isolated adolescents appraised hazardous situations as less risky following total isolation (Thomas et al., 2025). This change in risk perception occurred without changes in susceptibility to peer influence, suggesting that isolation primarily alters internal risk evaluation rather than responsiveness to social norms.

Access to virtual interaction during isolation partially buffered these effects. Although loneliness still increased, this effect was less pronounced than during total isolation, while reductions in mood were comparable (see Fig. 5). Virtual interaction mitigated some of the effects of isolation on reward processing and risk perception, but increases in threat learning persisted. This suggests that access to social media can satisfy some social needs, but it may not fully substitute for in-person interaction, particularly for threat hypervigilance.

Taken together, these findings indicate that even short-term social isolation leads to changes across cognitive systems: reward processes become more responsive, threat systems more vigilant and risk evaluation more permissive. While these changes may have adaptive value in motivating reconnection or enhancing self-protection, they may be maladaptive in contexts where isolation is imposed or long-term. The evidence from animal models described above suggests that prolonged social isolation can induce changes in stress and reward systems that increase susceptibility to anxiety and addictive behaviours, potentially through heightened stress reactivity and reward sensitivity.

More broadly, the evidence reviewed throughout this paper highlights how adolescent behaviour emerges from the interaction between ongoing neurocognitive development and the social and environmental contexts in which development takes place. These developmental characteristics are relevant to understanding offending during adolescence and raise questions about how youth justice systems should respond to young people whose behaviour occurs within a period of substantial neurocognitive and social change. The following section considers how these developmental characteristics can inform more effective approaches to preventing offending and promoting rehabilitation.

## 6 Section 4: Developmental cognitive neuroscience and youth justice

The evidence reviewed in the preceding sections points to three broad implications for youth justice. First, prolonged neuroplasticity indicates that adolescents retain a substantial capacity for learning and positive behavioural change. Second, because adolescents are especially sensitive to their social environment, peer influence can drive decision-making, including criminal behaviour, yet can also be harnessed to support rehabilitation. Third, evidence on social isolation underscores the role of supportive social connections in adolescence, showing that isolating young people can be counterproductive. Drawing on the developmental cognitive neuroscience reviewed above, this final section argues for evidence-based youth justice systems that replace punitive measures with developmentally appropriate legal thresholds and rehabilitative frameworks.

It has been argued that the developmental characteristics of the adolescent brain, described in Section 1 above, reduce criminal culpability, justifying a shift away from strictly punitive measures and toward rehabilitative approaches that harness ongoing neuroplasticity (Cavanagh, 2022, Monahan et al., 2009, Scott et al., 2018, Steinberg, 2009, Steinberg, 2013, Steinberg and Cauffman, 1996, Steinberg and Scott, 2003). In the US, developmental scientists, legal scholars and learned societies have been instrumental in translating research on brain development into practical court guidance, framing adolescence as a period of unique vulnerability and developmental opportunity. The influence of this translational work on legal policy has been substantial. Specifically, evidence of adolescent neuroplasticity and capacity for adaptive change played a pivotal role in landmark US Supreme Court decisions. In Roper v. Simmons (2005), which abolished the death penalty for juveniles (under 18), as well as Graham v. Florida (2010) and Miller v. Alabama (2012), which limited mandatory life sentences without parole for minors, the Court explicitly recognised that adolescents possess diminished maturity, a higher vulnerability to negative influences, a greater capacity for change and a greater potential for rehabilitation, compared with adults (Aspinwall et al., 2012).

Recently, the Criminal Court of Appeal for England and Wales acknowledged neurodevelopmental research demonstrating that the adolescent brain continues to mature up to the age of 25, concluding that: *It is categorically wrong to set about the sentencing of children and young people as if they are ‘mini-adults’. An entirely different approach is required.* (R v. Z.A., 2023). This has fundamentally altered the framework for assessing youth culpability and sentencing proportionality.

## 7 The minimum age of criminal responsibility

**Fig. 6:**
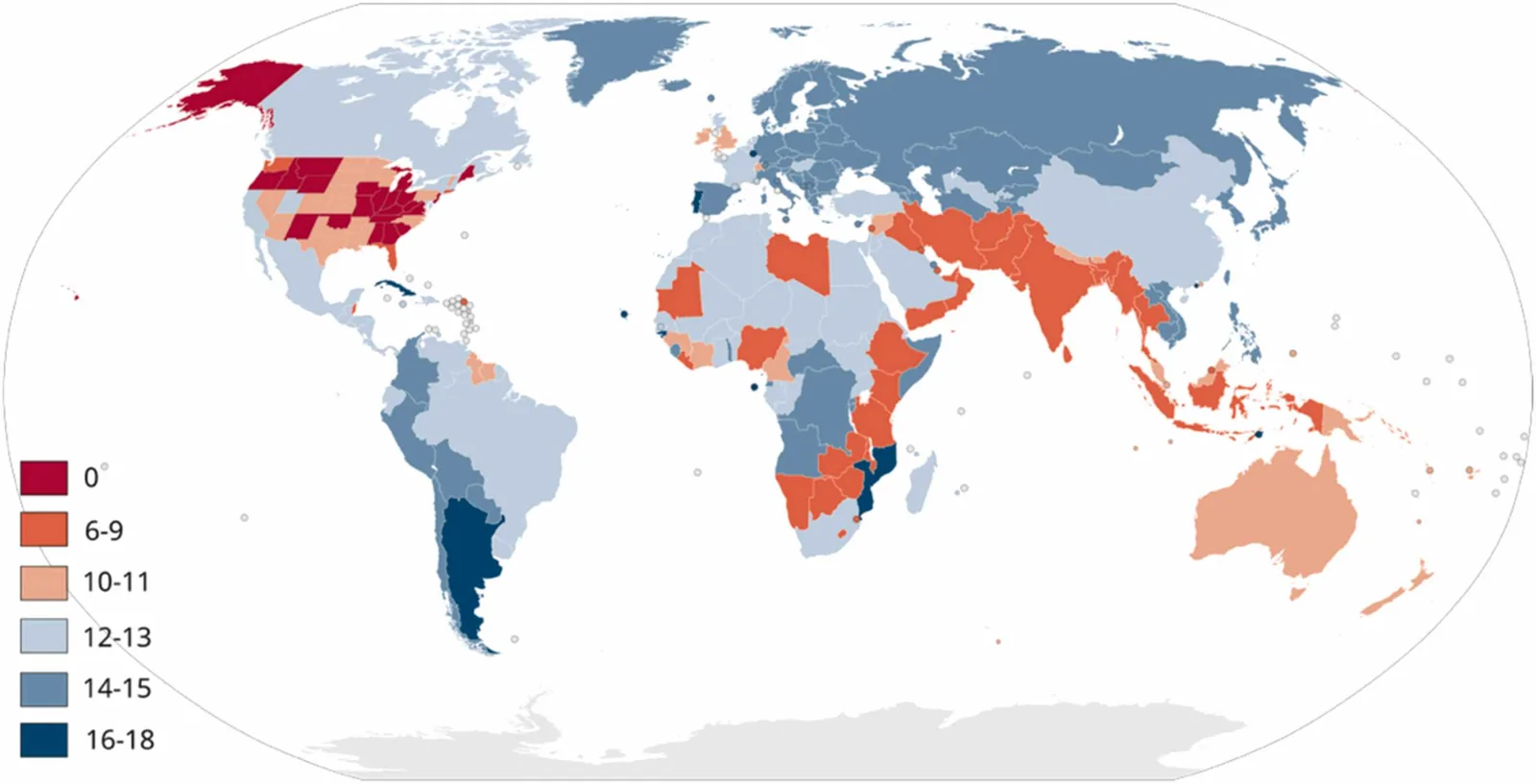
Global variation in the minimum age of criminal responsibility. Countries are shaded by age category, with warmer colours indicating lower ages and cooler colours indicating higher ages. From https://en.wikipedia.org/wiki/Age_of_criminal_responsibility based on data collated by the Child Rights International Network.

This neurodevelopmental evidence should be considered in debates surrounding the minimum age of criminal responsibility, defined as the youngest age at which a person can be held legally responsible for a crime. The minimum age of criminal responsibility is often shaped by social and political factors, leading to significant global variability (see Fig. 6). While many countries set the minimum age of criminal responsibility between 12 and 18 years, some jurisdictions, such as many U.S. states, have no established minimum age. Over the past two decades, several U.S. states have passed laws raising the age of criminal responsibility to 18, in part based on evidence that the brain continues to develop (Cavanagh, 2022). In the UK, the minimum age of criminal responsibility is notably low, set at 10 in England, Wales and Northern Ireland, and 12 in Scotland.

As described in Section 1, an average 10-year-old’s brain is still rapidly developing and far from being fully mature. Based partly on the neuroscientific evidence, coalitions of academics, clinicians and legal experts have advocated for raising the minimum age of criminal responsibility across the UK to align with other European nations (United Nations Committee on the Rights of the Child, 2023; ; The Bar Council, 2026). Similar arguments have gained traction in the US (Casey et al., 2022). This position is supported by the United Nations Committee on the Rights of the Child, which recommends a minimum age of at least 14 (United Nations Committee on the Rights of the Child, 2019). The United Nations has repeatedly criticised the UK for maintaining one of the lowest ages of criminal responsibility in Europe (United Nations Committee on the Rights of the Child, 2023). Despite these international standards and expert recommendations, UK policy has remained largely unchanged. This resistance is often attributed to high-profile cases of violent youth crime that dominate public discourse, reinforcing the perception that children are capable of adult-level criminality and making policymakers wary of the potential electoral backlash (House of Commons Briefing paper, 2016).

**Fig. 7:**
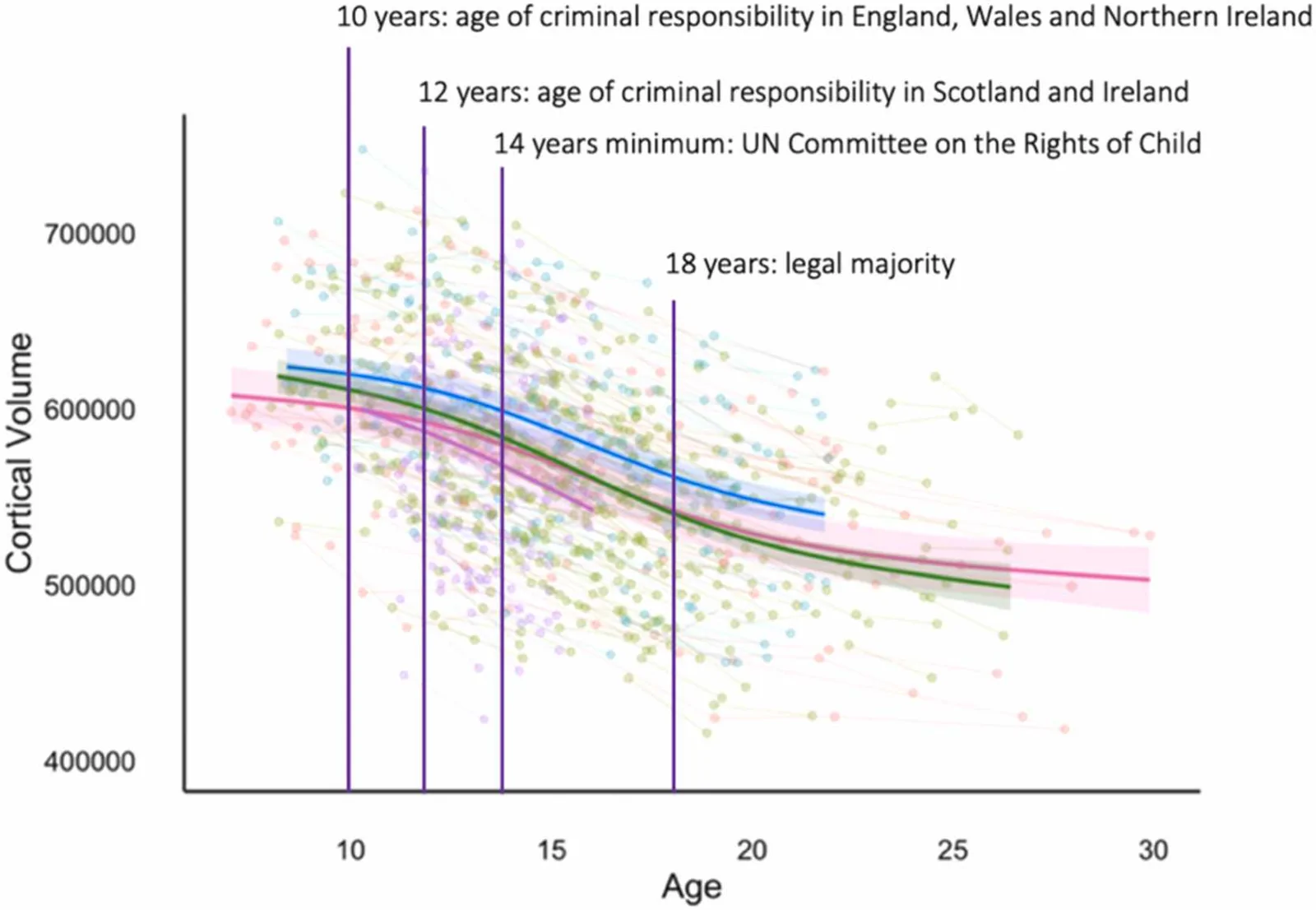
Age-related changes in cortical volume and minimum age of criminal responsibility. Scatterplot show cortical volume across age. Vertical lines indicate key legal thresholds in the UK and Ireland: 10 years (minimum age of criminal responsibility in England, Wales and Northern Ireland), 12 years (Scotland and Ireland), 14 years (minimum recommended by the UN Committee on the Rights of the Child) and 18 years (legal majority in most countries). Adapted from Tamnes et al. (2017).

While neuroscientific evidence provides important context for these debates, it also highlights the inherent challenges of setting rigid legal age boundaries. Longitudinal cohort studies demonstrate that the brain undergoes substantial development during this period, and it is clear that the average brain is far from mature by age 10. However, population-level averages obscure substantial individual variability. As shown in Fig. 7, there is significant variation in both the baseline (intercept) and the rate of change of cortical volume with age (slope) across different individuals (Foulkes and Blakemore, 2021, Mills et al., 2014).

It is partially due to the neuroscientific evidence showing that the brain undergoes continuous development into the late twenties that adolescence has been redefined as spanning from ages 10–24 (Sawyer et al., 2018). Yet, this biological timeline stands in conflict with legal frameworks that impose a rigid, binary transition into full adult responsibility at age 18 in most jurisdictions. This divergence leaves young adults vulnerable to punitive sentencing during the final stage of brain development.

However, translating neuroscience into precise legal age boundaries presents significant challenges. There is no single age at which the human brain can be universally classified as ‘adult,’ as neurodevelopment is a continuous process without sharp transition points, marked by substantial individual variation. Furthermore, a degree of experience-dependent plasticity is retained across the entire lifespan (Marzola et al., 2023).

Thus, while neuroscience provides a framework for understanding the developmental trajectories and plasticity of the adolescent brain, it cannot independently prescribe exact legal thresholds or define a single age of maturity. Legal policies must integrate neuroscientific evidence with broader societal values and evidence from complementary disciplines, including developmental psychology research on adolescent cognitive, emotional and social development.

## 8 The social context of adolescent offending

As described in Section 2, developmental psychology research demonstrates that a young person may understand that a behaviour is wrong, yet still lack the cognitive control required to resist acting on impulse. This capacity for self-control is further compromised in emotionally charged environments (Somerville et al., 2011), and the mere presence of peers can disrupt an adolescent’s ability to regulate impulsive actions (Blakemore, 2018). Indeed, the link between peers and adolescent and criminal behaviour has been documented for over a century (Breckinridge and Abbott, 1912, Shaw and & Moore, 1931). Adolescents are strongly influenced by the behaviour and attitudes of peers (Ahmed et al., 2020, Moffitt, 1993, Kim and Fletcher, 2018) and are more likely to engage in risky, antisocial or criminal behaviour when accompanied by peers than when alone (McGloin and Thomas, 2019, Reiss and Farrington, 1991).

This peer-driven increase in antisocial behaviour in adolescence is conceptualised in Moffitt’s (1993) developmental taxonomy, which proposes that most adolescent offending is a temporary, socially normative response rather than a reflection of a deep-seated pathology (Moffitt, 1993, Moffitt et al., 2002). Moffitt proposes it is driven by the ‘maturity gap', the delay between biological puberty and the attainment of socio-economic autonomy. To signal independence, adolescents mimic the risk-taking behaviours of rebellious or older peers. As a context-dependent mechanism of social adaptation, this behaviour typically desists as individuals transition into adult roles and responsibilities (Moffitt, 2018).

Because most adolescent offending is a temporary phase, the justice system’s response during this window can either facilitate natural desistance or inadvertently trap young people in persistent criminal trajectories. Early encounters with legal authorities can influence ‘legal socialisation’, the process through which young people form enduring attitudes toward the law (Fine et al., 2022, Fine and van Rooij, 2021). When young people experience procedurally unfair treatment from authorities, they are predisposed to view the law as illegitimate, which predicts future reoffending (Cavanagh, 2022). Furthermore, these negative attitudes spread rapidly through peer networks, as adolescents whose friends have been arrested report lower institutional trust (Fine et al., 2016). Overly punitive responses do not only alienate individual young people, they create a shared ethos of systemic cynicism that can entrench the very criminal behaviour the system seeks to deter.

This risk of alienation is particularly acute under legal doctrines that interpret adolescent peer dynamics as collective criminal intent. In many jurisdictions, mechanisms of secondary liability, such as joint enterprise in the UK or the *Pinkerton* doctrine and felony murder rules in the US, allow criminal responsibility to extend beyond the principal offender (Hulley and Young, 2025). As outlined in Section 2, developmental research demonstrates that adolescents are particularly vulnerable to peer influence in group settings and often comply with peer norms to avoid social exclusion. When legal frameworks treat peer co-presence or compliance as calculated complicity, they disproportionately penalise young people for developmental vulnerabilities. These findings highlight a need for legal frameworks to account for adolescents' heightened sensitivity to peer dynamics when evaluating culpability in cases involving shared or secondary liability.

Furthermore, these individual and interpersonal vulnerabilities do not exist in a vacuum; they interact with broader structural and contextual inequalities. Broader systemic factors such as structural inequities, such as socioeconomic deprivation, racialised policing and pervasive surveillance, play a role in determining which young people are funnelled into the legal system. When the neurocognitive vulnerabilities of adolescence, such as heightened reward seeking and peer sensitivity, collide with these inequitable structural conditions, the risk of justice system involvement is amplified.

Institutional disciplinary practices frequently miscalculate adolescent social needs during intervention. Isolation is routinely used as a form of disciplinary punishment, for example, via the use of school exclusions, school isolation booths and custodial solitary confinement (Lee, 2016). However, as described in Section 3, brief periods of isolation can alter adolescent cognitive and emotional processing, heightening reward-seeking, elevating threat sensitivity and reducing risk perception (Thomas et al., 2025, Tomova et al., 2025, Towner et al., 2024). Rather than suppressing antisocial behaviour, punitive isolation risks exacerbating behavioural difficulties and emotional distress.

Instead, the developmental social sensitivity that amplifies risky behaviour can be harnessed for prosocial redirection. Moving away from punitive isolation allows for rehabilitative models that leverage adaptive neuroplasticity and incorporate positive peer relationships. Evidence indicates that youth justice programmes can reduce reoffending by cultivating positive peer relationships and using mentoring to encourage prosocial behaviour and mutual accountability (South et al., 2014). For example, the Arches Transformative Mentoring programme in the US demonstrated a significant reduction in reconvictions among high-risk adolescents (Lynch et al., 2018). Similarly, Canada’s Positive Peer Culture programme has been linked to lower reoffending rates (Brendtro and Caslor, 2019), while the EQUIP programme, which combines peer support with cognitive-behavioural training, has shown substantial reductions in recidivism across multiple settings (Gibbs et al., 1995, Leeman et al., 1993). Together, these findings suggest that positive peer support is a promising strategy for promoting rehabilitation among justice-involved young people.

While the developmental findings reviewed in this paper have implications across law and policy, their scope must be qualified by certain limitations. First, controlled laboratory conditions lack the ecological validity required to replicate the multifaceted stressors of real-world justice environments. Second, sampling disparities exist between the community participant samples included in the experimental studies described in this paper and justice-involved populations, who frequently exhibit compounded vulnerabilities in mental, behavioural and relational health, and are disproportionately from disadvantaged backgrounds. Furthermore, a limitation of the neurocognitive data is its heavy reliance on samples from Western, educated, industrialised, rich and democratic (WEIRD) nations, which restricts the extent to which these findings can be assumed to generalise globally across diverse cultural and socio-economic contexts. Yet, while these psychological and environmental variances mean lab-derived insights may not be directly generalisable to all youth in the criminal justice system, they do not diminish the core value of the neurodevelopmental framework.

## 9 Conclusion

Developmental cognitive neuroscience has redefined adolescence as a sensitive period of adaptation rather than mere volatility, and offers an empirical foundation for justice system reform. The heightened neuroplasticity that characterises this period of life endows the brain with a unique capacity for change, providing a compelling rationale for shifting away from punitive sanctions and towards rehabilitation. Simultaneously, the social reorientation of adolescence renders young people particularly vulnerable to peer influence, a dynamic that can amplify risk-taking and antisocial behaviour out of need to avoid social exclusion, but can also be harnessed to drive prosocial redirection. Conversely, enforced social isolation alters cognitive and affective processing, possibly exacerbating the very vulnerabilities the justice system seeks to address. Together, these findings challenge simplistic portrayals of adolescents as reckless, demonstrating that their decisions and actions are often driven by a powerful need for social connection and peer affiliation. Accounting for these developmental factors, alongside structural inequalities and legal socialisation, requires a shift toward a legal framework of mitigated culpability, where legal policies align with empirical evidence to foster environments that mitigate risk while supporting adolescents’ capacity to adapt, reform and thrive.

## CRediT authorship contribution statement

**Sarah-Jayne Blakemore:** Writing – review & editing, Writing – original draft, Conceptualization.

## Declaration of Competing Interest

The author declares the following financial interests which may be considered as potential competing interests: She has provided expert advice to UK charities, legal organisations in the UK and USA, and the UK Government. She is the author of two books on the brain, education and learning. She has given talks in state and independent schools, at education conferences, and for education organisations and other public, private and third-sector organisations, and is a member the UK Bar Council working group on the minimum age of criminal responsibility.

## Data Availability

No data was used for the research described in the article.
